# Non-random distribution of gastric cancer susceptible loci on human chromosomes

**DOI:** 10.17179/excli2018-1425

**Published:** 2018-08-17

**Authors:** Ghazale Mahjoub, Mostafa Saadat

**Affiliations:** 1 Department of Biology, College of Sciences, Shiraz University, Shiraz, Iran

## ⁯

Dear Editor,

It has been well established that many molecular alterations are involved in the etiology of cancers. Genetic studies indicated that gastric cancer (GC) has significant heritability in human populations (Graham et al., 1994[10]; Drăghicescu et al., 1998[6]; Gao et al., 2011[7]) through different molecular and genetics features (Gigek et al., 2017[8]). In order to find the genetic elements involved, many studies investigated the association of genetic variations of a wide range of candidate genes. Meta-analyses studies have shown significant associations of many polymorphisms of candidate loci with the risk of GC at least in a specific ethnic group.

Numerous data revealed the non-randomness distribution of genes on human chromosomes (Hecht 1988[13]; Lima-de-Faria et al., 1991[22]; Mouchiroud et al., 1991[33]; Saccone et al., 1996[45]; Musio et al., 2002[34]; Rafiee et al., 2008[40]). Previously our study group has reported that polymorphic loci which were associated with the risk of breast cancer (Saify and Saadat, 2012[46]), Alzheimer's disease (Saadat, 2016[44]), schizophrenia (Saadat, 2013[42]), Parkinson's disease and multiple sclerosis (Saadat, 2014[43]) are non-randomly dispersed on human chromosomes. Based on our knowledge, there is no published data about randomness of distribution of the GC susceptible loci on human chromosomes. Therefore the present study was carried out.

A literature database (PubMed) was searched for relevant studies (the last search was updated in February 2018). The following search terms were used: Gastric cancer, meta-analysis, and genetic polymorphism. The search was limited to articles published in English. There were significant associations between genetic polymorphisms of 64 genes and the risk of GC in at least one human ethnic groups. Table 1(Tab. 1) (References in Table 1: Chang et al., 2014[1]; Chen et al., 2015[2]; Cheng et al., 2015[3]; Dai et al., 2015[4]; Deng et al., 2014[5]; Gong et al., 2016[9]; Han et al., 2012[11]; He et al., 2013[12]; Hua et al., 2014[14]; Jia et al., 2017[16]; Kuang et al., 2014[17]; Li et al., 2015[20]; Li et al., 2016[18]; Li et al., 2017[19]; Liang et al., 2018[21]; Liu et al., 2011[23]; Liu et al., 2011[24]; Liu et al., 2014[25]; Liu et al., 2014[26]; Lu et al., 2012[27]; Ma et al., 2013[29]; Ma et al., 2013[30]; Ma et al., 2017[28]; Mo et al., 2016[31]; Mocellin et al., 2015[32]; Namazi et al., 2018[35]; Pabalan et al., 2015[36]; Peng and Xu, 2015[37]; Qi et al., 2016[38]; Qin et al., 2017[39]; Ribeiro et al., 2017[41]; Shen et al., 2014[47]; Shi et al., 2014[48]; Shi et al., 2017[49]; Shi et al., 2018[50]; Tian et al., 2012[52]; Wang et al., 2013[53]; Wang et al., 2014[54]; Wang et al., 2015[56]; Wang et al., 2015[57]; Wang et al., 2016[55]; Wu et al., 2015[58]; Xie et al., 2017[59]; Xu et al., 2014[61]; Xu et al., 2014[63]; Xu et al., 2015[62]; Xu et al., 2018[60]; Yadav et al., 2018[64]; Yan et al., 2013[65]; Yang et al., 2014[66]; Yang et al., 2016[67]; Ye et al., 2017[68]; Yu et al., 2014[69]; Zhang et al., 2012[72]; Zhang et al., 2013[71]; Zhang et al., 2015[74]; Zhang et al., 2016[70]; Zhang et al., 2016[73]; Zhao et al., 2014[75]; Zhou et al., 2014[76]) summarized these studies. 

To evaluate the randomness/non-randomness distribution of GC susceptible loci on chromosomes, the statistical method of Tai and his colleagues (1993[51]) was used. The relative width of human chromosomal band was determined using the diagram of the International System for Chromosome Nomenclature (ISCN, 1981[15]). P-values less than 0.05 were considered as significant differences.

Analysis revealed that the 64 susceptible loci were distributed non-randomly on chromosome segments. The 1q22 (P<0.001), 2q14.1 (P<0.001), 5q31-q33 (P<0.001), 6p12-p21 (P<0.001), 10q23 (P<0.001), 11q13-q22 (P=0.025), 12q13.13 (P<0.001), 16q22.1 (P<0.001), 17q21-q25 (P<0.001), 19p13 (P=0.025) and 19q13 (P=0.025) were bearing higher numbers of GC susceptible loci. The human chromosome segments 6p12-p21, 17q21-q25, and 11q13-q22 were bearing seven (*IL-17A*, *IL-17F*, *VEGFA*,* CDKN1A*, *TNF-α*, *LTA*, and *HspA1B*), five (*TP53*, *BRCA1*, *NME1*, *ACE*, *TIMP-2*, and *BIRC5*) and four (*GSTP1*, *CCND1*, *MMP7*, and *MMP1*) GC susceptible genes, respectively.

The current findings have two significant aspects:

1. Distribution of the susceptible genes is not random throughout the human chromosomes.
2. The present findings help investigators to design a mass screening test tool for finding high risk persons to GC using the genetic polymorphisms in above-mentioned segments.

Previously it has been reported that human chromosome segments 10q23.3-q24.3, 16q13-q22.1, 17q12-q23, 19q13.1-q13.4, 22q11.2-q13.2 were significantly bearing breast cancer susceptible loci (Saify and Saadat, 2012[46]). Comparing with the present findings, the segments 10q23, 16q22.1, 17q12-q23, and 19q13 revealed significant associations with both gastric and breast cancers.

## Acknowledgements

The authors are indebted to Dr. Maryam Ansari-Lari for critical reading of the manuscript. This study was supported by Shiraz University, Iran. 

## Conflict of interest

None.

## Figures and Tables

**Table 1:**
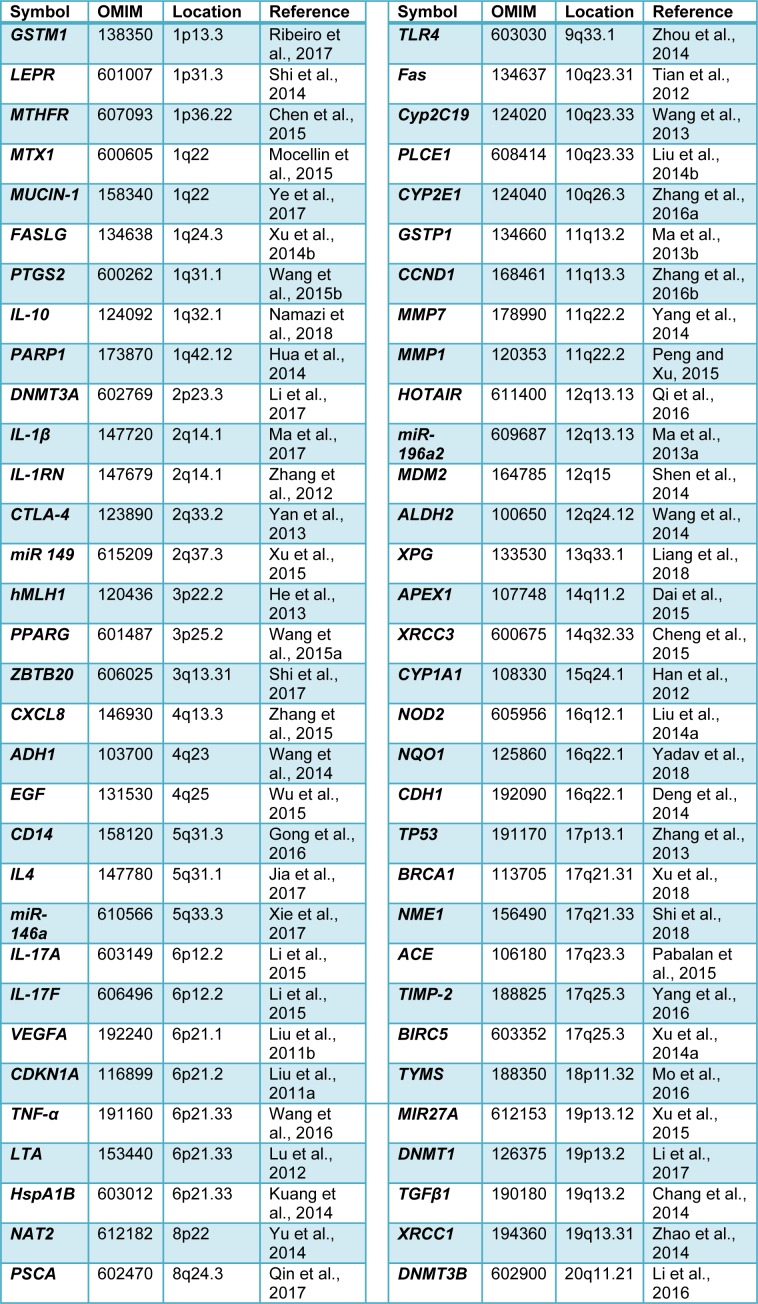
List of polymorphic loci associated with susceptibility to gastric cancer
